# redPATH: Reconstructing the Pseudo Development Time of Cell Lineages in Single-cell RNA-seq Data and Applications in Cancer

**DOI:** 10.1016/j.gpb.2020.06.014

**Published:** 2021-02-17

**Authors:** Kaikun Xie, Zehua Liu, Ning Chen, Ting Chen

**Affiliations:** 1 Institute for Artificial Intelligence, State Key Lab of Intelligent Technology and Systems, Department of Computer Science and Technology, Tsinghua University, Beijing 100084, China; 2 Tsinghua-Fuzhou Institute of Digital Technology, Beijing National Research Center for Information Science and Technology, Tsinghua University, Beijing 100084, China; 3 Center for Computational and Integrative Biology, Massachusetts General Hospital, Harvard Medical School, Boston, MA 02114, USA; 4 Department of Molecular Biology, Massachusetts General Hospital, Harvard Medical School, Boston, MA 02114, USA; 5 Broad Institute of Massachusetts Institute of Technology and Harvard, Cambridge, MA 02142, USA

**Keywords:** Single-cell pseudotime reconstruction, Consensus Hamiltonian path, Cell differentiation, Cell proliferation, Cell development and diseases

## Abstract

The recent advancement of single-cell RNA sequencing (scRNA-seq) technologies facilitates the study of cell lineages in developmental processes and cancer. In this study, we developed a computational method, called redPATH, to reconstruct the pseudo developmental time of cell lineages using a consensus asymmetric Hamiltonian path algorithm. Besides, we developed a novel approach to visualize the trajectory development and implemented visualization methods to provide biological insights. We validated the performance of redPATH by segmenting different stages of cell development on multiple neural stem cell and cancer datasets, as well as other single-cell transcriptome data. In particular, we identified a stem cell-like subpopulation in malignant glioma cells. These cells express known proliferative markers, such as *GFAP*, *ATP1A2*, *IGFBPL1*, and *ALDOC*, and remain silenced for quiescent markers such as *ID3*. Furthermore, we identified *MCL1* as a significant gene that regulates cell apoptosis and *CSF1R* for reprogramming macrophages to control tumor growth. In conclusion, redPATH is a comprehensive tool for analyzing scRNA-seq datasets along the pseudo developmental time. redPATH is available at https://github.com/tinglabs/redPATH.

## Introduction

Developmental research at a single-cell level has been supported by flow cytometry and imaging methods over the past few decades. Three fundamental questions of interest include how individual cells develop into different cell types and tissues, how cells function, and the underlying mechanism in gene regulations. Such cell development processes have yet remained significantly obscure [1]. Recent advances in single-cell RNA sequencing (scRNA-seq) technologies [2] have enabled us to characterize the transcriptome of individual cells. This allows us to study the subtle difference in heterogeneous cell populations. For example, single-cell analyses in tumors, immunology, neurology, and hematopoiesis have led to new and profound biological findings [3], [4], [5], [6], [7], [8].

Specifically, for glioblastoma (GBM), single-cell analysis reveals the functionality of the tumor microenvironment in GBM. The relationships among microglia/macrophages, malignant cells, oligodendrocytes, and T cells have been uncovered, confirming previous biological conclusions [3], [4], [5]. Notably, glioma-associated microglia/macrophages (GAMs) are known to regulate tumor growth, adversely changing their functionality under normal conditions [9], [10], [11], [12], [13]; recent research has targeted GAMs to re-activate their anti-tumor inflammatory immune response to suppress tumor growth [13]. In addition, previous studies have identified potential markers (such as *CSF1R*) for the reprogramming of GAMs; however, it acquired resistance over time and resumed vigorous tumor growth [14], [15].

Many algorithms have been developed to study cell development processes, including cell differentiation and cell proliferation, by inferring a pseudotime trajectory at the single-cell level for both snapshot data and multiple time-point data. A recent review [16] compared multiple state-of-the-art methods for developmental trajectory inference including Monocle2 [17], TSCAN [18], and SCORPIUS [19], and for cell cycle processes such as reCAT [20]. Popular trajectory tools also include Seurat, DPT, Wishbone, and numerous others [21], [22], [23], [24], [25]. Both Monocle2 and TSCAN assume a free branching structure of cell fate development, whereas SCORPIUS assumes a linear development. Most of the existing methods would have two main steps, linear or non-linear dimensionality reduction, followed by trajectory inference.

Monocle2 [17] uses an unsupervised feature selection named ‘dpFeature’. It first selects the differentially expressed genes among unsupervised clusters of cells. Then a principal graph is learned via a reverse graph embedding (RGE) algorithm, ‘DDRTree’, where it reflects the structure of the graph in a much lower-dimensional space. Then Monocle2 calculates a minimum spanning tree (MST) on the distance of projection points to find a connected trajectory. Pseudotime is inferred by projecting cells onto the MST trajectory.

TSCAN [18] takes into consideration the dropout event. The raw gene expression is first processed by gene clustering to gain an average gene expression level. Since many of the gene clusters are highly correlated, TSCAN reduces the dimensionality using principal component analysis (PCA). Then MST is applied to cell cluster centroids, which are inferred from the reduced space to form a trajectory. Finally, each cell is projected onto the MST trajectory to obtain the pseudotime.

SCORPIUS [19] is a fully unsupervised trajectory inference method. First, it calculates the Spearman’s rank correlation between cells and defines an outlier metric for each cell. Then multi-dimensional scaling (MDS) is applied to the correlation distance matrix to learn a low-dimensional representation of each cell. An initial principal curve is then calculated as the shortest path between the *k*-means (*k* set to 4) cluster centers. The principal curve is then learned iteratively by projecting the cells onto the curve.

Traditional trajectory inference analyses would remove cell cycle effects through the removal of cycling genes. However, the cell cycle process and cell differentiation process seem to be coupled according to recent research, especially in the development of neural stem cells (NSCs) [26]. Within the sub-ventricular zone (SVZ), it is estimated that 80% of adult NSCs undergo symmetric differentiation, and 20% undergo symmetric proliferation with little evidence of asymmetric divisions. To date, only one computational method, CycleX [27], attempts to decipher such a relationship between the two developmental processes.

**Figure 1:**
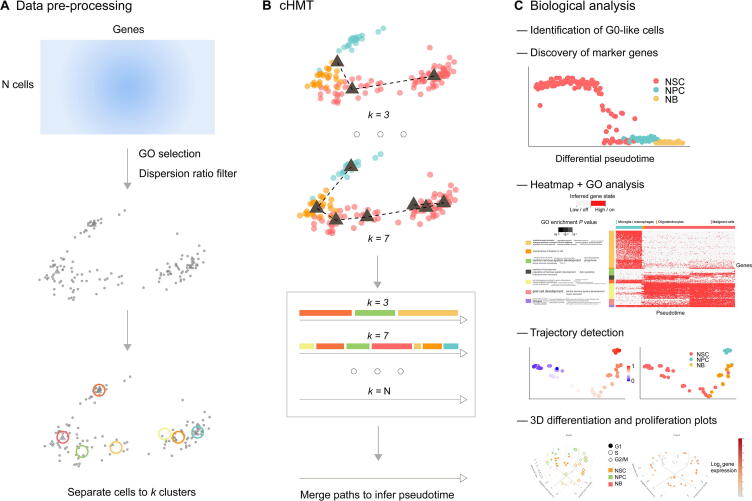
**Overview of redPATH** The pipeline of the algorithm and analysis included in redPATH. **A.** and **B.** Algorithm schematic illustration of redPATH comprising data pre-processing (A) and the cHMT algorithm (B). **C.** The main biological analysis functions included in redPATH. GO, Gene Ontology; cHMT, consensus Hamiltonian path; NSC, neural stem cell; NPC, neural progenitor cell; NB, neuroblast.

In this study, redPATH successfully recovered the pseudotime of the differentiation process and discovered some unique genes along with cell development (Figure 1). The performance and stability of redPATH were validated and compared with multiple state-of-the-art methods, showing its consistency in explaining marker gene expression changes. Here, we first implemented a consensus Hamiltonian path (cHMT) algorithm to reconstruct the pseudotime of a linear differentiation process. We proposed to model the differentiation process between cells using an asymmetric measure (Kullback-Leibler distance). The linear development assumption has importance in studies of more differentiated lineages at later stages of development, allowing us to study the relationship between differentiation and proliferation more clearly.

Additionally, we developed an approach to decipher and combine multiple Hamiltonian path solutions into a transition matrix to visualize the trajectory (linear or branched) developmental trend. We implemented downstream analyses and visualization methods to provide biological insights into the developmental processes. In particular, redPATH is incorporated with reCAT to visualize the relationship between differentiation and cell cycle within the cell development of neural cells. Finally, we analyzed glioma datasets with a new perspective, uncovering a subpopulation within malignant cells.

## Method

### Overview of redPATH

Figure 1 shows that redPATH consists of three main steps: data pre-processing, pseudotime inference, and biological analysis. There are two main challenges in pseudotime inference in single-cell transcriptome data: the curse of dimensionality and the high level of noise, which together can severely affect the performance of pseudotime inference. Hence, we first performed the gene filtering steps to reduce the dimensionality and then developed a consensus algorithm to overcome the high level of noise.

There are two main assumptions for redPATH. First, we assumed the higher similarity between cells within the same cell type or the same developmental stage than those between different states. Second, we assumed a linear and directional development of cells. Despite the linear assumption in our model, we can utilize multiple linear Hamiltonian path solutions to detect both linear and branching trajectories.

### Data pre-processing

The pre-processing step includes standard normalization procedures using existing methods such as edgeR and DEseq2 if the gene expression matrix is not normalized [28], [29], [30], [31]. We took the log_2_ [transcripts per kilobase million (TPM) + 1] or  log_2_ [fragments per kilobase million (FPKM) + 1] for expression value. Then we used the Gene Ontology (GO) database [32], [33], [34] to select genes that are associated with the following ontologies (hereby referred to as GO genes): “cell development”, “cell morphology”, “cell differentiation”, “cell fate”, and “cell maturation”. Note that the selection of genes still includes a portion of cell cycling genes.

The selected ontologies are ones that are closely related to cell development. We then filtered out the selected genes using a dispersion ratio. The dispersion ratio is simply a ratio of the mean over its standard deviation. We set the cut-off to 10 to retain at least a few hundred genes. For each gene *j*, we calculated the ratio denoted by *disp*_*j*_ using the following formula:

(1) $$\mathrm{dis}p_{j}=\frac{\sum_{i}{\left(c_{\mathrm{ij}}-\mu_{j}\right)}^{2}}{\left(N-1\right)\mu_{j}}$$

where *c*_*ij*_ is the *j*-th gene expression value in the *i*-th cell and *μ*_*j*_ is the average gene expression value for the *j*-th gene over all cells. In all the analyses, the cut-off is set by default to $\mathrm{dis}p_{j}⩾10$. This feature selection procedure reduces dimensionality from ten thousands of genes to a few hundred.

### cHMT

Let X be the gene expression matrix of N cells (rows) by M selected GO genes (columns). We want to infer an N by one vector denoting the pseudotime of each cell. This problem can be re-modeled as a Hamiltonian path problem. There are two assumptions: cells are similar within a particular cell type, and the differentiation process is linear. Although many heuristic solutions have been developed to solve this NP-complete problem, most produce inconsistent results due to locally optimal solutions [35]. To overcome this difficulty, we developed a cHMT solution to infer the pseudotime. The algorithm consists of the following main steps:

**Table:** 

**Algorithm 1 cHMT**
**Input:** X(N, M)
**for***k* = 3 **to** N **do**
Cluster X into *k* groups of cells
Generate X′(*k*, M) by taking the average over *k* clusters
Compute Hamiltonian path solution for each *k*
**end for**
Merge each path solution to produce final solution
**Output:** A N by one pseudotime vector

Intuitively, we first inferred a rough pseudotime ordering of large clusters of cells, and then gradually refined the solution with the increase of *k*.

The clustering method in step 1 is inspired by spectral clustering and SCORPIUS [19]. Briefly, the Spearman correlation distance matrix is first calculated as the pairwise distance between two cell vectors $x_{i},x_{j}$:

(2) $$D\left(x_{i},x_{j}\right)=\frac{C\left(x_{i},x_{j}\right)+1}{2}$$

where *x*_*i*_ and *x*_*j*_ are both *m*-dimensional vectors of the GO gene expression, and *C*() denotes the Spearman correlation value. Then using the N by N correlation distance matrix, we applied double centering to normalize the matrix. Finally, a simple hierarchical clustering is applied for $k\in \left[3\ldots N\right]$.

Next, a Hamiltonian path problem is solved for each value of *k*. In this context, the solution is a path that visits each cell cluster or cell while minimizing the total distance. Hence, the definition of the distance function is vital to the final solution. A naïve cost function would be the Euclidean distance between the cluster centers. However, to better model the biological mechanism, we proposed an asymmetric distance measure, namely the Kullback-Leibler distance (KL-distance), or more often referred to as KL-divergence [36]. KL-divergence simply measures the difference between the two distributions. In this scenario, we made a pairwise comparison between each *m*-dimensional cell vector: *x*_*i*_ (when *k* = N) or cluster averaged vector (when *k* < N). In the following notations, the cell vector will encompass the cluster averaged vector in general.

(3) $$d_{\mathrm{KL}}\left(x_{i}\left|\right|x_{j}\right)=\sum x_{i}*n\frac{x_{i}}{x_{j}}$$

**Supplementary Figure S1:**
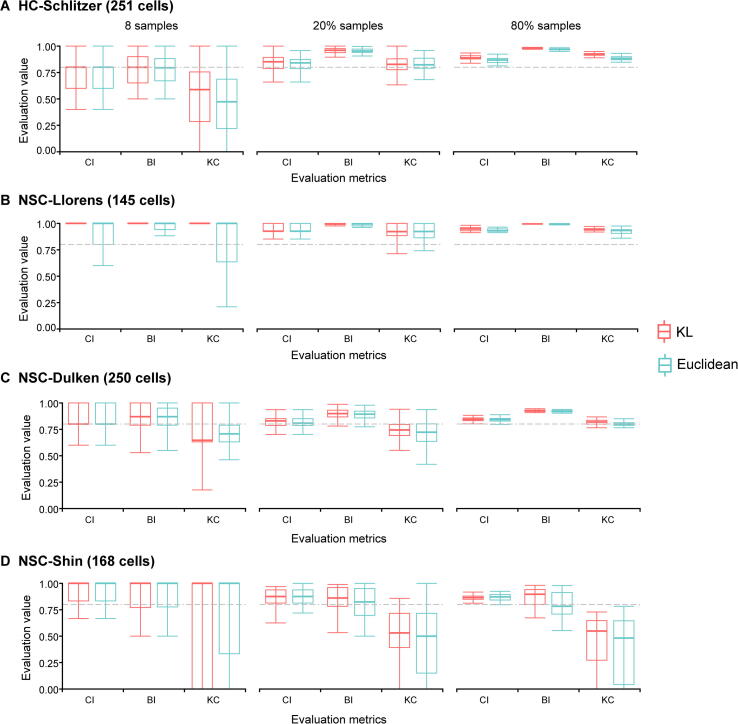
**Comparison of distance measures’ effect on redPATH**. Evaluation results of CI, BI, and KC are shown for HC-Schlitzer (**A**), NSC-Llorens (**B**), NSC-Dulken (**C**), and NSC-Shin (**D**), respectively. We compared the difference in the results of redPATH using the Euclidean distance and KL distance. Fifty different samples were taken for 8 cells, 20% of cells, and 80% of cells respectively for each column. A brute force Hamiltonian path is performed on 8 sampled cells, and redPATH is performed on the remaining two cases. Boxplot represents the standard quantile values based on the results from 50 random samples. CI, change index; BI, bubble sort index; KC, Kendall correlation.

Equation (3) shows the calculation of the KL-distance for two different distributions, *i.e.*, from *x*_*i*_ to *x*_*j*_, with each representing an *m*-dimensional distribution of gene expression. The vice versa direction would result in a different value. The intuition here is that the differentiation process is directional and irreversible. Hence, given a more differentiated cell, the distance for it to reverse back to a less differentiated cell should be penalized. Although we cannot be sure of which cell is more or less differentiated, the KL-distance metric gives a small directional restriction in this sense. Direct comparisons of the performance of KL-distance and Euclidean distance are presented in File S1 and Figure S1.

After calculating the pairwise distance between each cell vector, we turned to the modeling of a Hamiltonian path problem. We first defined the problem as a graph G = (V, E) where V is the list of vertices, and E represents the edges. Each vertex corresponds to a cell vector $x_{i}=\left(x_{i1},x_{i2},\ldots ,x_{\mathrm{iM}}\right)$. The edge weight between each vertex is calculated by $d_{\mathrm{KL}}\left(x_{i}\left|\right|x_{j}\right)$ and $d_{\mathrm{KL}}\left(x_{j}\left|\right|x_{i}\right)$ as defined in Equation (3). The goal here is to find the shortest path that visits each vertex or cell once.

Next, we developed an O(n^2^)-time heuristic algorithm with a simple modification for the arbitrary insertion algorithm [35] for the Hamiltonian path problem. Note that the Hamiltonian path problem is a classic NP-hard problem, so no algorithm guarantees an optimal solution in any case. Briefly, our heuristic algorithm considers the asymmetric property of our data and modifies the insertion cost calculation. The main structure of the algorithm remains the same, but we provided a new perspective using asymmetrical distances directly. Details of the modified algorithm are provided in File S1. We performed the modified algorithm multiple times, ensuring the quality and robustness of each Hamiltonian path solution. Additionally, we developed a novel approach to find the initial start and endpoint, increasing the probability of finding the optimal global solution.

To overcome the instability of the Hamiltonian path solutions and refine cell heterogeneity at single-cell resolution, we proposed a consensus approach similar to reCAT. An advantage of the proposed algorithm is that it will automatically discover the start and end cells of the path. First, we inferred a reference Hamiltonian path using the enumerated results of five different paths obtained from *k* = 3 to 7 (by default). Since there are two possible starting points of each solution, we then calculated the pairwise correlation between each of the four paths and their respective reverse, yielding ^5^C_2_ = 10 additive correlation scores and 2^5^ = 32 total comparisons. We took the ordering direction of the best combinations determined by the best correlation score. All five paths are merged to give a reference path by projecting onto the space of [0,1] and taking the average. The base path is then normalized by feature scaling once again. Hence, the direction of the path is determined by the reference path. Subsequently, for each of the following Hamiltonian paths, *k* = 7 to N, the Spearman correlation of each path and its reverse is compared with the reference path. Finally, after adjusting the direction, each path is then merged to the reference path to obtain our final pseudotime. The cHMT ordering is essentially obtained by sorting the pseudotime values.

### Trajectory identification using multiple Hamiltonian paths

Furthermore, we developed an approach to visualize the trajectory (linear or branched) development of cells. Intuitively, redPATH recovers a linear pseudotime by merging multiple path solutions to a single path, which naturally merges branching situations. We hypothesized that we can detect the branching trajectory by observing the detailed transition of cells in each of the merged solutions.

The main idea is to transform multiple Hamiltonian path solutions into a transition probability matrix followed by PCA visualization. Given *p* Hamiltonian path solutions, we recorded all the transitions in each path *pi* and construct an N by N transition matrix. For instance, if N = 3 and the *pi*-th solution is 2–3–1, we add a probability value for the transitions 2–3 and 3–1. We sum the transition probabilities together until all Hamiltonian path solutions are recorded.

### Biological analysis

We can identify key genes or gene modules specific to the differentiation process with the Hamiltonian path ordering. To quantify the expression changes over the pseudotime, we used two statistical measures: maximal information coefficient (MIC) and distance correlation (dCor) [37], [38]. Compared to the standard Pearson or Spearman correlation coefficients, these measures are more robust and range from 0 to 1. The scores are calculated for all of the genes and ranked accordingly. We selected genes that exceed a threshold of 0.5 for downstream analysis. However, the downside of these measures is that they cannot determine a positive or negative correlation.

Then we designed a simple hidden Markov model (HMM) to infer two (or possibly three) hidden states of each gene. The two hidden states represent an on/highly expressed or off/lowly expressed state in each cell. The observed variable is simply the gene expression value. In this model, we assumed a univariate Gaussian distribution over the two hidden states.

The model is initialized with $N\left(\mu_{h1},\sigma_{h1}\right)$ and $N\left(\mu_{h2},\sigma_{h2}\right)$ where the mean and standard deviation are estimated with the sorted observed gene expression values. Then the transition probability is inferred using the Baum-Welch algorithm [39]. Subsequently, we implemented the Viterbi algorithm to infer the hidden states of the gene in each cell. The inferred states are then clustered using hierarchical clustering and visualized through a heatmap to provide an overall understanding of the gene expression changes over the developmental process. The gene clusters are further analyzed using GOsummaries [40] and PANTHER [41], which provide some biological insights into the gene modules.

### Coupling the differentiation and cell cycle processes

In order to identify the relationship between cell differentiation and proliferation, we incorporated reCAT and redPATH to visualize their relationship. One of the challenges faced in analyzing the cell cycle is the removal of G0 cells. To date, there is no known algorithm to identify G0 cells. Here, we developed a novel approach using statistical tests to identify the G0 cells before further analysis.

**Supplementary Figure S2:**
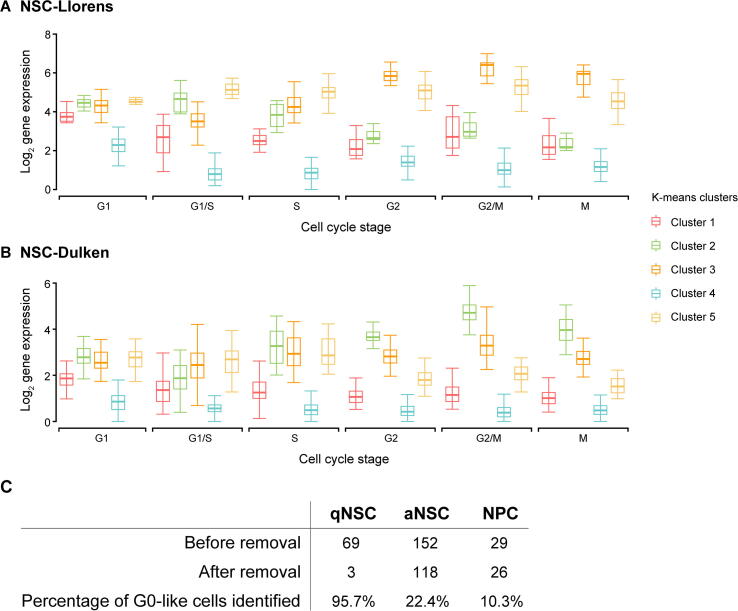
**Distribution of cycling scores and number of identified G0 cells**. The distribution of cell clusters in each of the six cell cycle scores (namely G1, G2, S, M, G1S, and G2M) was plotted for NSC-Llorens (**A**) and NSC-Dulken (**B**). We identified cluster 4 as the lowest cluster. ANOVA test was performed to confirm that it is significantly lower than all other clusters in all six scores. C. shows the percentage of G0-like cells removed in NSC-Dulken compared to the ground truth. qNSC is considered to be G0-like. qNSC, quiescent neural stem cell.

The intuition for the developed approach is that cell cycling genes tend to be inactive in G0 cells and that such cells are in a resting phase. Hence, we hypothesized that G0-like cells will have the lowest cycling expression. We first transformed the gene expression matrix into average expression values for each of the following six mean cell cycle scores, G1, S, G1/S, G2, M, and G2/M. We used the annotations from Cyclebase (adapted from reCAT) to calculate the average scores. Then we applied *k*-means with *k* set to 5 (*i.e.*, G0, G1, S, G2, and M stages) to the mean scores. We performed the pairwise analysis of variance (ANOVA) test for each of the mean scores to test for the least expressed group. The criterion is set such that the identified group must be significant (*P* < 0.001) in all of the six mean scores compared with the remaining groups. We validated the results on a couple of datasets where the G0 cells are known (File S1; Figure S2).

After removing G0 cells, we inferred the pseudo differentiation and cycling time for each cell using reCAT and redPATH, respectively. Then we produced 3D spiral plots as an attempt to visualize their relationship. Briefly, the pseudotime of reCAT is projected onto a circle as the X and Y axes, and then the differentiation time is plotted on the Z-axis. Marker genes are used to depict the gradual change of the cell types in each dataset.

### Evaluation metrics

To quantitatively assess the pseudo temporal ordering, we used four metrics to compare pseudotime results. There are limitations to evaluating the accuracy of orderings because the delicate ordering within each different cell type remains unknown. The only available information is the cell type labeling obtained from biological experiments, which may contain some bias due to technical noise during biological experiments. Using the cell type information, we developed the change index (CI) and bubble sort index (BI). We further applied the Kendall correlation (KC) and pseudo-temporal ordering (POS) score to evaluate the reconstructed pseudotime.

For illustration, we used an example of a linear development of neural cells. A linear development of the neural system in the SVZ is defined from quiescent neural stem cells (qNSCs) to activated neural stem cells (aNSCs), then differentiating into neural progenitor cells (NPCs). In other words, we assumed that we have three stages of development, ordering from qNSC to aNSC to NPC.

The first metric, CI, was adopted from reCAT [20]. Assuming the number of states is *ns* (which is 3 in our example), we calculated the number of state changes, *s*, after re-ordering the cells. Then we calculated CI as CI = 1 – (*s* – *ns* – 1)/(N – *ns*) where N is the total number of cells. Hence, a temporal ordering that completely resembles the actual labeling of cell types would have a value of 1 and the worst case of 0.

From experimental results, we found that CI may be inaccurate when a large subset of a particular cell type is grouped but inserted within another cell type of development. Hence, we designed a second metric, BI, to evaluate the re-ordered cells. The intuition is inspired by the number of steps *s* taken to re-sort the cells. Specifically, this is the required number of steps to switch adjacent cells to reach the correct ordering. BI has better stability than CI. The number of steps *s* is then divided by S, the number of steps taken to sort the worst-case scenario (*i.e.*, the reverse of the correct ordering), to produce BI. Generally, BI results in higher values in the range of [0,1].

Thirdly, we also used the Kendall correlation coefficient to evaluate our pseudotime. Both Spearman and Kendall correlation would work better than the Pearson correlation in this case due to the consideration of ranking in these two methods. Additionally, the POS score is also adapted from TSCAN [18] to evaluate the performance.

## Results

### Validation and evaluation of redPATH

We first validated the intuition of redPATH and then compared its performance with current state-of-the-art algorithms. We extensively evaluated the performance on three neuronal datasets [6], [7], [42], one mouse hematopoietic dataset [8], one human hematopoietic dataset [43], and three embryonic time point datasets [44], [45], [46]. The further downstream analysis included recent glioma datasets [5] to uncover underlying mechanisms behind cancerous cells. All analyzed datasets are listed in Table 1.

**Table 1:** **Description of analyzed datasets**




*Note*: NSC, neural stem cell; HC, hematopoietic cell; ESC, embryonic stem cell; HSC, hematopoietic stem cell; mESC, mouse embryonic stem cell; mCV, mouse cardiovascular cell; mGas, mouse gastrulation cell; MGH, Massachusetts General Hospital; WHO, World Health Organization; hHSC, human hematopoietic stem cell.

For the neuronal datasets of Dulken and Llorens-Bobadilla, both studies look at the development of NSCs in the SVZ. In contrast, Shin’s data were obtained from the subgranular zone (SGZ). The development lineage is quite clear where qNSCs become aNSCs and differentiate into NPCs, which finally differentiate into neuroblasts (NBs) or neurons. The hematopoietic data look at the development of dendritic cells near the end of the lineage. The macrophage and dendritic cell precursors (MDPs) differentiate into common dendritic cell precursors (CDPs) and give rise to pre-dendritic cells (preDCs). An important question of interest is how differentiation and proliferation processes are regulated within these different cells. We explored this question in the later parts of this paper, which discusses the incorporation of reCAT and redPATH to provide a simple exploratory analysis.

#### *Quantitative evaluation of redPATH*

**Figure 2:**
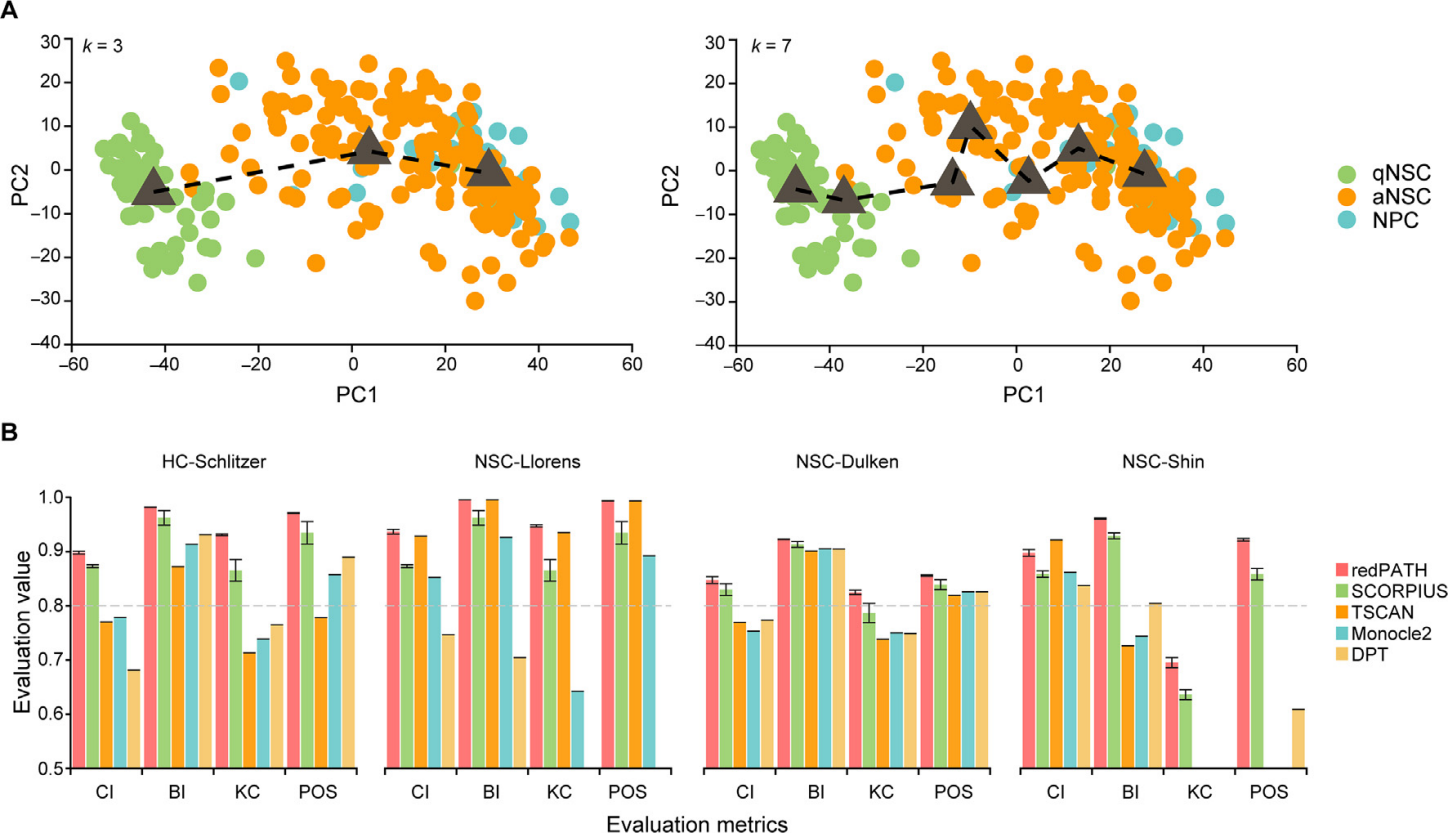
**Validation of redPATH** **A.** The Hamiltonian path solution by projecting each cell and cluster center onto scaled and centered PCs for *k* = 3 (left) and *k* = 7 (right). The dark gray triangles represent the cluster centers, and the dotted line reflects the Hamiltonian path. The color of each cell reflects the cell type label. **B.** Performance evaluation of different algorithms on four single-cell datasets using CI, BI, KC, and POS. Bar plots are colored by the algorithms used, and the leftmost bar (in red) represents redPATH. The error bar represents the 95% confidence interval based on 20 runs of the algorithm. Missing bars in the plots represent an evaluation value of less than 0.5. PC, principal component; CI, change index; BI, bubble sort index; KC, Kendall correlation; POS, pseudo-temporal ordering score; qNSC, quiescent neural stem cell; aNSC, activated neural stem cell.

First of all, we verified that modeling the single-cell trajectory as a Hamiltonian path problem is a valid approach. Figure 2A shows the developmental process across cells aligned by the Hamiltonian path for *k* = 3 and *k* = 7 clusters. This sets the foundation for redPATH. Assuming the order of the development progression is correct, the ordering is refined by combining the paths of larger *k* and thereby obtaining a stable solution.

We then compared redPATH with SCORPIUS, TSCAN, Monocle2, and DPT. In Figure 2B, redPATH consistently shows the best performance across all the scores for the three neuronal datasets and one hematopoietic dataset.

**Supplementary Figure S3:**
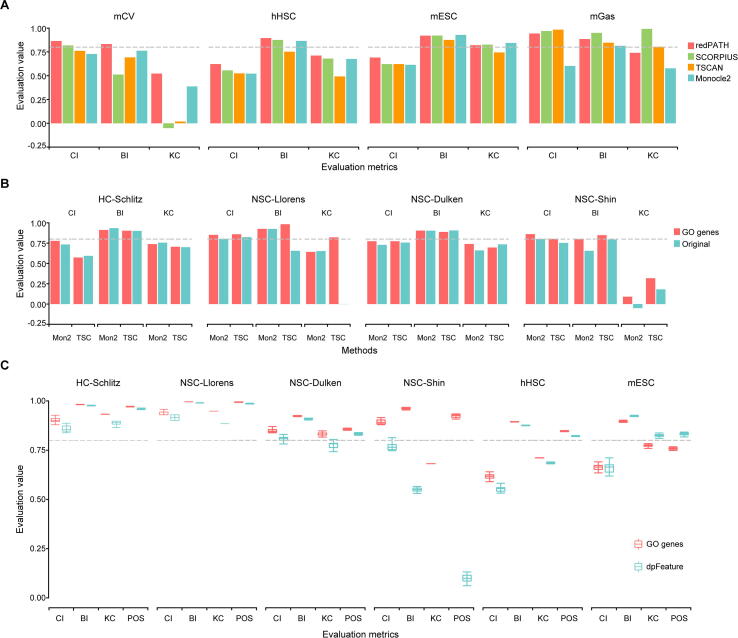
**Evaluating the robustness of redPATH**. **A**. Quantitative evaluation of multiple time point data. The performance of each algorithm was evaluated on mCV, hHSC, mESC, and mGas, respectively. The gray dotted line marks the 0.8 threshold for the evaluation metrics of CI, BI, and KC. **B**. Evaluation of the effect of feature selection steps. The performance of both Mon2 and TSC was compared using different feature selection methods on four datasets. We used the original feature selection for Monocle2 and TSCAN (referred to as ‘Original’ in the plot); both methods were compared against GO gene selection (from redPATH). **C**. Performance of redPATH using different gene selections. We compared GO genes with dpFeature on six different datasets. The boxplots represent the standard quantile distribution based on 20 runs of the algorithm. Mon2, Monocle2; TSC, TSCAN; GO, Gene Ontology.

We used the same filtered input expression profiles (the selected GO genes) for each of the algorithms. SCORPIUS claims to be robust when using all the genes without gene selection, but the performance drops across all datasets when using the full gene expression matrix. Note that NSC-Llorens performs quite well overall, partially because the data are sequenced at a much deeper length. The error bars in Figure 2B represent a 95% confidence interval based on 20 runs of both SCORPIUS and our algorithm. Furthermore, redPATH (CI: 0.69, BI: 0.92, KC: 0.82) is on par with SCORPIUS (CI: 0.62, BI: 0.92, KC: 0.84) on a multi-time point dataset (mESC) with ten cell types (Figure S3A). The outperformance of CI also proves its capability to analyze time point data as well as snapshot data. We also evaluated additional multi-time point datasets (Figure S3A).

**Figure 3:**
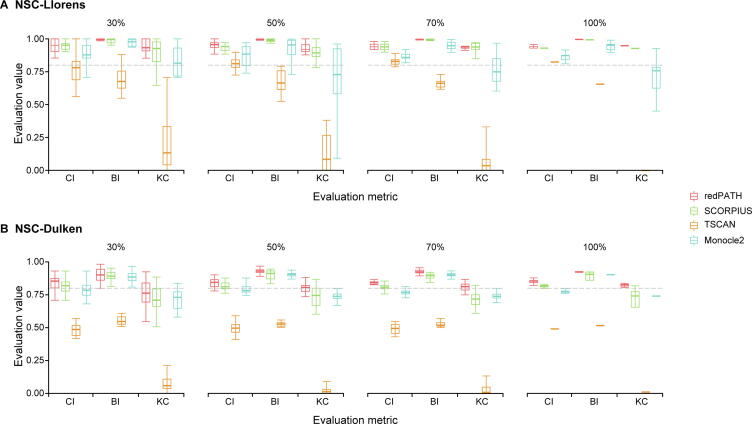
**Robustness analysis on algorithms** **A.** 30%, 50%, 70%, and all cells were sampled from the NSC-Llorens dataset. **B.** 30%, 50%, 70%, and all cells were sampled from the NSC-Dulken dataset. The color represents the algorithm used. Each algorithm was evaluated for the same 20 subsamples using CI, BI, and KC. The boxplot represents the standard quantile range for the calculated values. The gray horizontal line denotes the 0.8 mark for the evaluation value.

The performance of many algorithms may be susceptible to cell subpopulation as well as different gene selections. In Figure 3, we presented the robustness of each algorithm on subsamples of cells. For both NSC-Llorens and NSC-Dulken, we sampled 30%, 50%, 70%, and 100% of all cells 20 times. As shown in Figure 3A, the evaluation of redPATH on all three metrics is relatively consistent and stable; we also observed a similar pattern in Figure 3B. A comparison of different gene selection approaches is provided in File S1 and Figure S3B. Additionally, we also compared the performance of redPATH with a different set of genes using dpFeature (Figure S3C).

##### *Subtle differences in inferred biological development*

**Figure 4:**
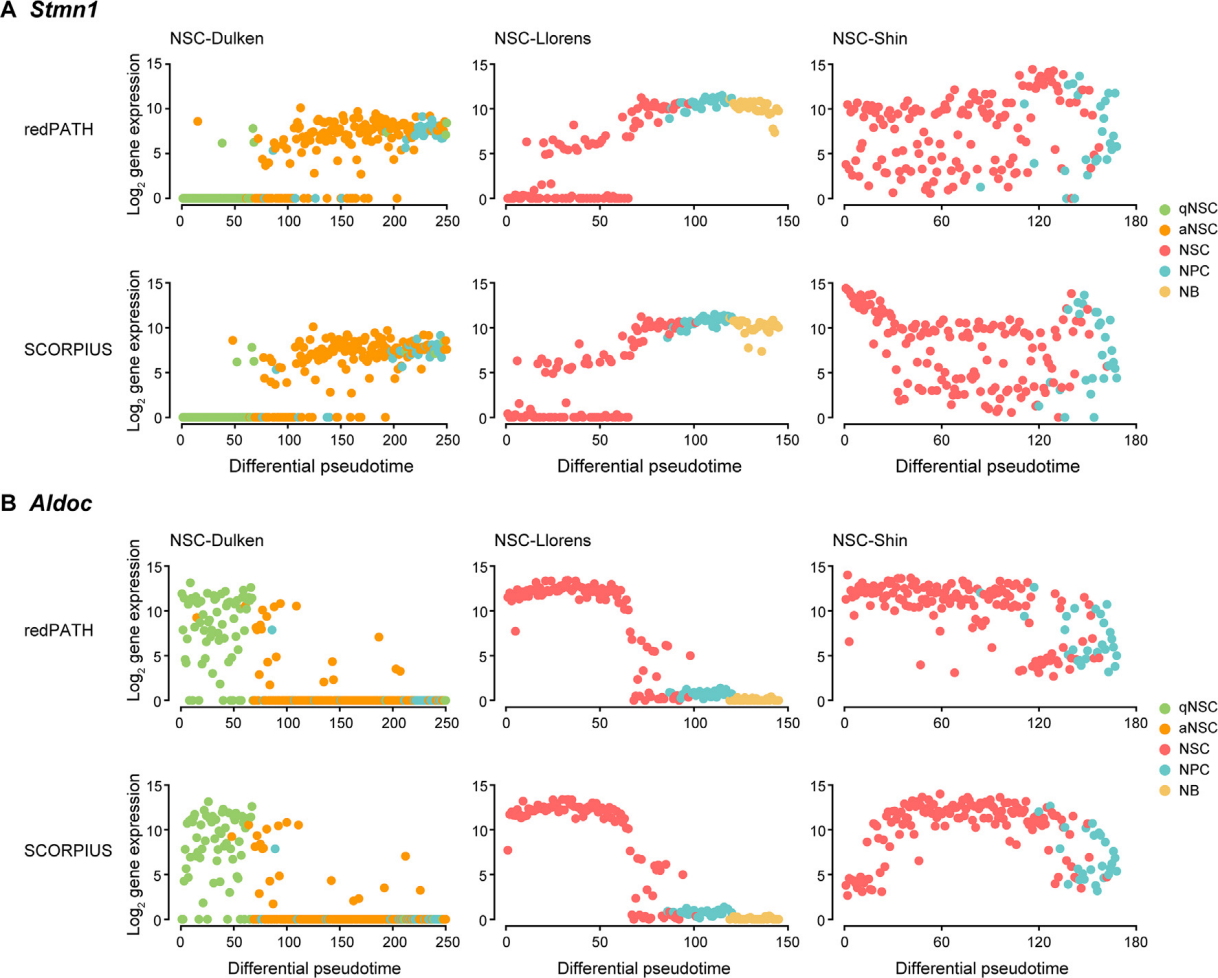
**Qualitative comparison on expression changes** **A.** Difference in gene expression trend for *Stmn1*. **B.** Difference in gene expression trend for *Aldoc*. Gene expression is plotted against the inferred pseudotime. Comparison is made across three neuronal datasets (NSC-Dulken, NSC-Llorens, and NSC-Shin separately for each column) using redPATH and SCORPIUS.

**Supplementary Figure S4:**
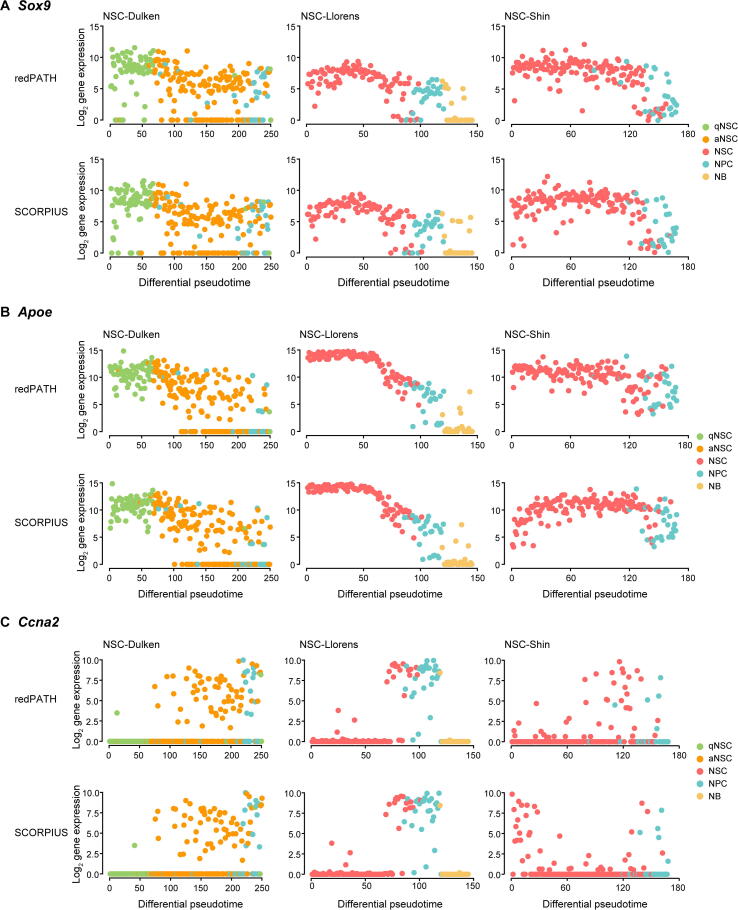
**Additional comparisons on NSC datasets**. The subtle differences in the inferred pseudotime are further compared to NSC marker genes (*Sox9*, *Apoe*) and a proliferative marker gene (*Ccna2*). We depict the difference in gene expression trend for *Sox9* (**A**), *Apoe* (**B**), and *Ccna2* (**C**) by plotting the gene expression against the inferred pseudotime. We compared three NSC datasets (NSC-Dulken, NSC-Llorens, and NSC-Shin separately for each column) using redPATH and SCORPIUS. NSC, neural stem cell.

Accounting for all the metrics across each dataset, SCORPIUS has a relatively better performance than the other methods. To further explore the differences in biological functions between redPATH and SCORPIUS pseudotime, we observed the developmental trends on some marker genes on all three neuronal datasets (Figure 4). *Stmn1* and *Aldoc* [42], [47], [48] are considered as marker genes for the differentiation of NSCs. *In vivo* experiments [42] have shown that *Stmn1* is highly expressed in NPCs but lowly expressed in NSCs; *Aldoc* is highly expressed in qNSCs and lowly expressed in aNSCs and NPCs. We compared the gene expression development inferred by redPATH and SCORPIUS due to their better overall performance. A comparison of additional marker genes is included in File S1 and Figure S4.

On both NSC-Dulken and NSC-Llorens datasets, the performance of redPATH is on par with SCORPIUS, and we observed no significant difference. However, the ordering of SCORPIUS clearly shows a different pattern on the NSC-Shin dataset compared to that on the other two neuronal datasets. Take the *Stmn1* gene as an example (Figure 4A), SCORPIUS starts with high expression (which is supposed to be lowly expressed at the start of the trajectory) and then decreases on the NSC-Shin dataset. This observation is different from the conclusion made from biological experiments. redPATH fits the developmental trend with relatively low expression at the beginning of the trajectory and shows consistency across datasets for the same cell type. We can observe that SCORPIUS tends to identify some bell-shaped trend, which could be explained by iteratively fitting principal curves in their algorithm. This observation can also be found for the *Aldoc* gene on the NSC-Shin dataset (Figure 4B). Here, redPATH proves to be robust across different datasets and correctly orders the developmental pseudotime in accordance with biological observations.

##### *Identifying trajectory development of cells*

**Figure 5:**
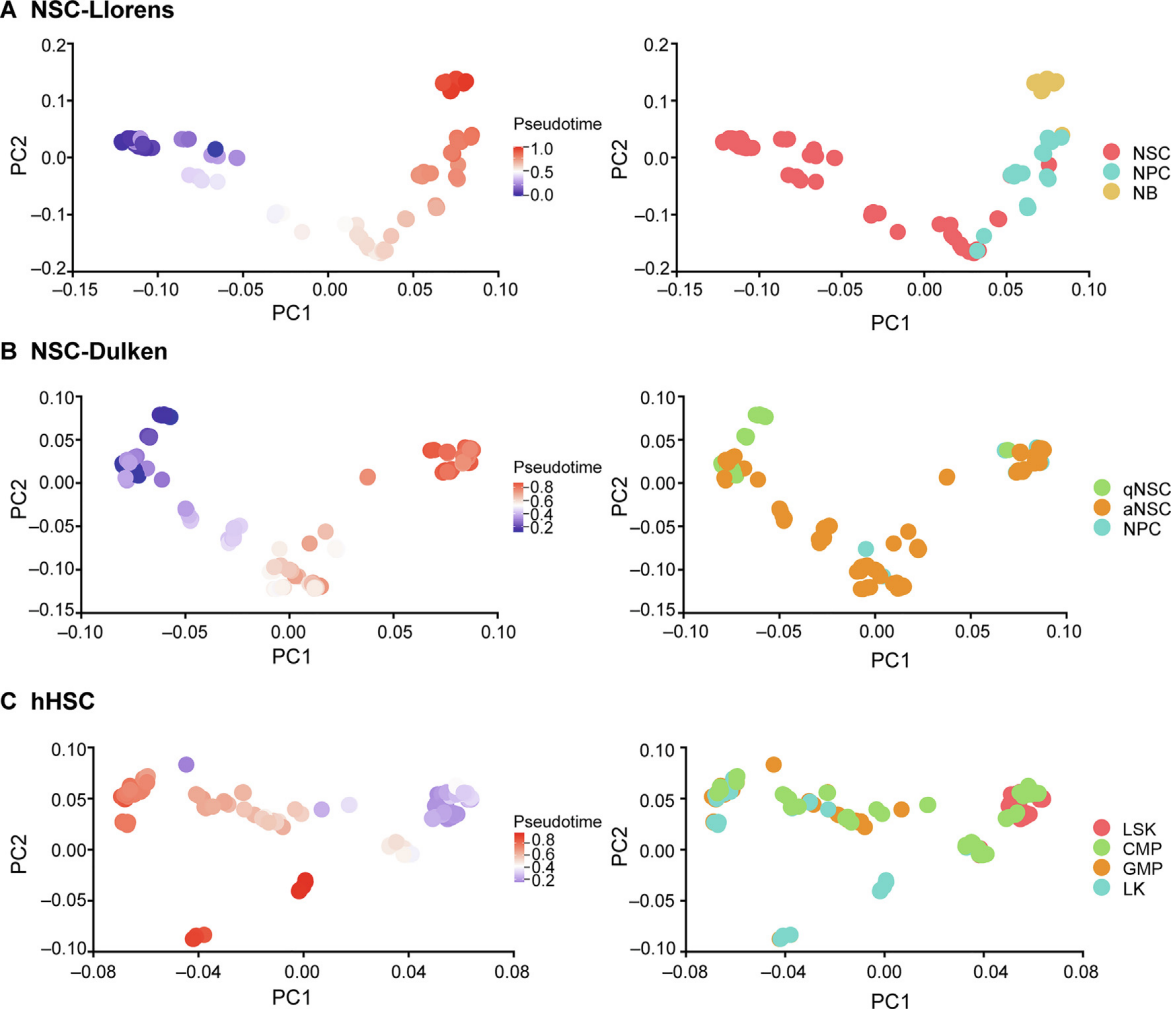
**Trajectory visualization** Visualization of the differentiation development process colored by pseudotime and cell-type information for NSC-Llorens (**A**), NSC-Dulken (**B**), and hHSC (**C**) datasets. Each point represents a cell in space, and PCA is performed on the calculated transition matrix. The left panel depicts the pseudotime of each cell, and the right shows the corresponding cell type information. hHSC, human hematopoietic stem cell; LSK, stem/multipotent progenitor cell (Lin^–^, Sca1^+^, c-Kit^+^); CMP, common myeloid progenitor cell; GMP, granulocyte monocyte progenitor cell; LK, LK CD34^+^ cell (Lin^–^, c-Kit^+^, CD34^+^); PCA, principal component analysis.

**Supplementary Figure S5:**
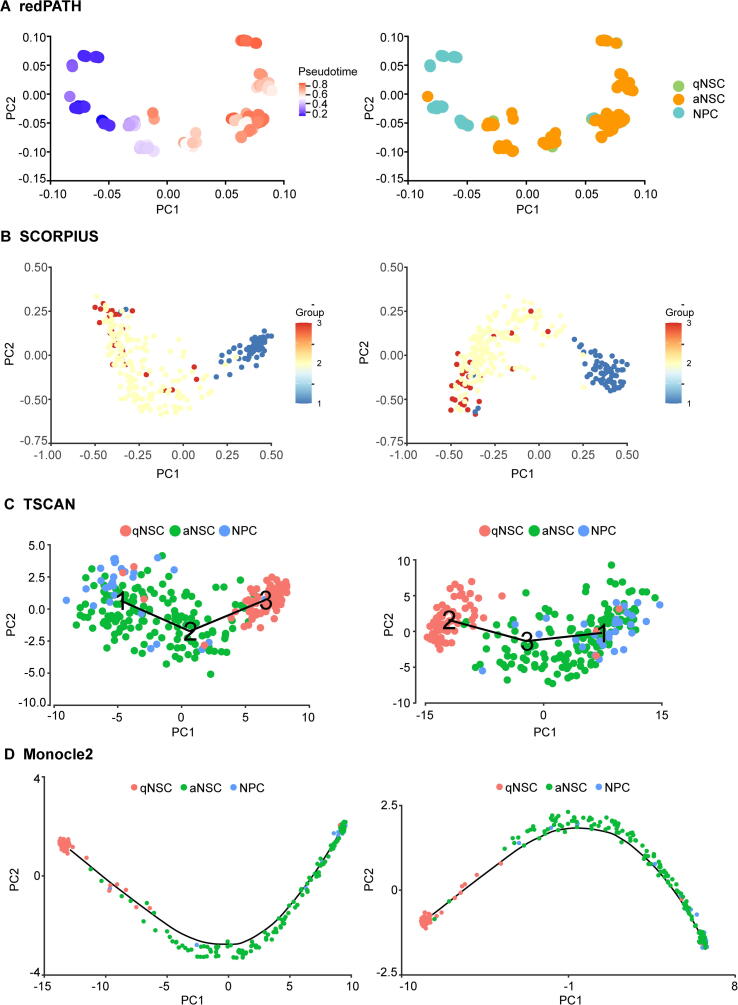
**Trajectory plots for NSC-Dulken**. Trajectory plots were generated for redPATH (**A**), SCORPIUS (**B**), TSCAN (**C**), and Monocle2 (**D**), respectively, using the default functions in their respective R packages. For redPATH, the left panel shows the pseudotime progression of cells, while the right denotes the cell type information. For SCORPIUS, TSCAN, and Monocle2, the left panel shows the trajectory obtained from using the default gene selection method in their respective package. On the right, the input expression is pre-processed by using the GO selected genes from redPATH.

**Supplementary Figure S6:**
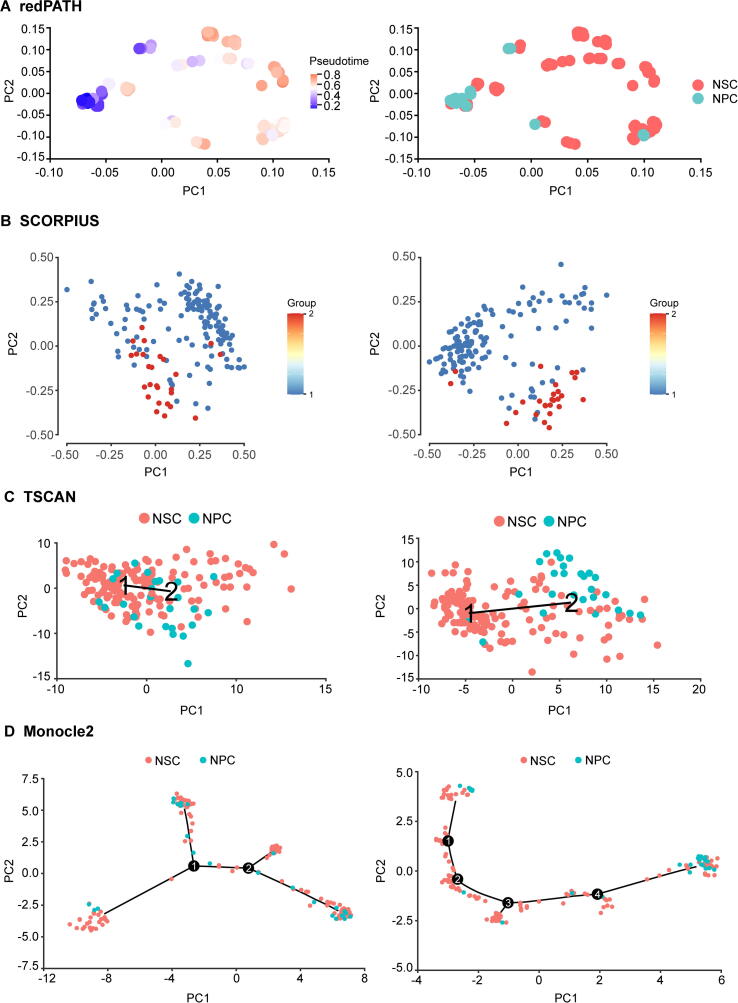
**Trajectory plots for NSC-Shin**. Trajectory plots were generated for redPATH (**A**), SCORPIUS (**B**), TSCAN (**C**), and Monocle2 (**D**), respectively, using the default functions in their respective R packages. For redPATH, the left panel shows the pseudotime progression of cells, while the right denotes the cell type information. For SCORPIUS, TSCAN, and Monocle2, the left panel shows the trajectory obtained from using the default gene selection method in the respective package. On the right, the input expression is pre-processed by using the GO selected genes from redPATH.

Utilizing the multiple Hamiltonian path solutions from redPATH, we can construct a cell transition matrix and visualize the developmental trend on a PCA plot (Figure 5). The trajectory plots are shown for two linear progression datasets, NSC-Llorens and NSC-Dulken, as well as a branching human hematopoietic stem cell dataset (hHSC). The progression in cells along the pseudotime reflects a linear development from NSC to NPC (Figure 5A and B). However, for the hHSC dataset, the PCA plot suggests a branching development of cells (Figure 5C), confirming the original discovery of binary cell fate decisions [49]. We further compared the trajectories produced by different algorithms in Figures S5 and S6.

### Coupling proliferation with differentiation

**Figure 6:**
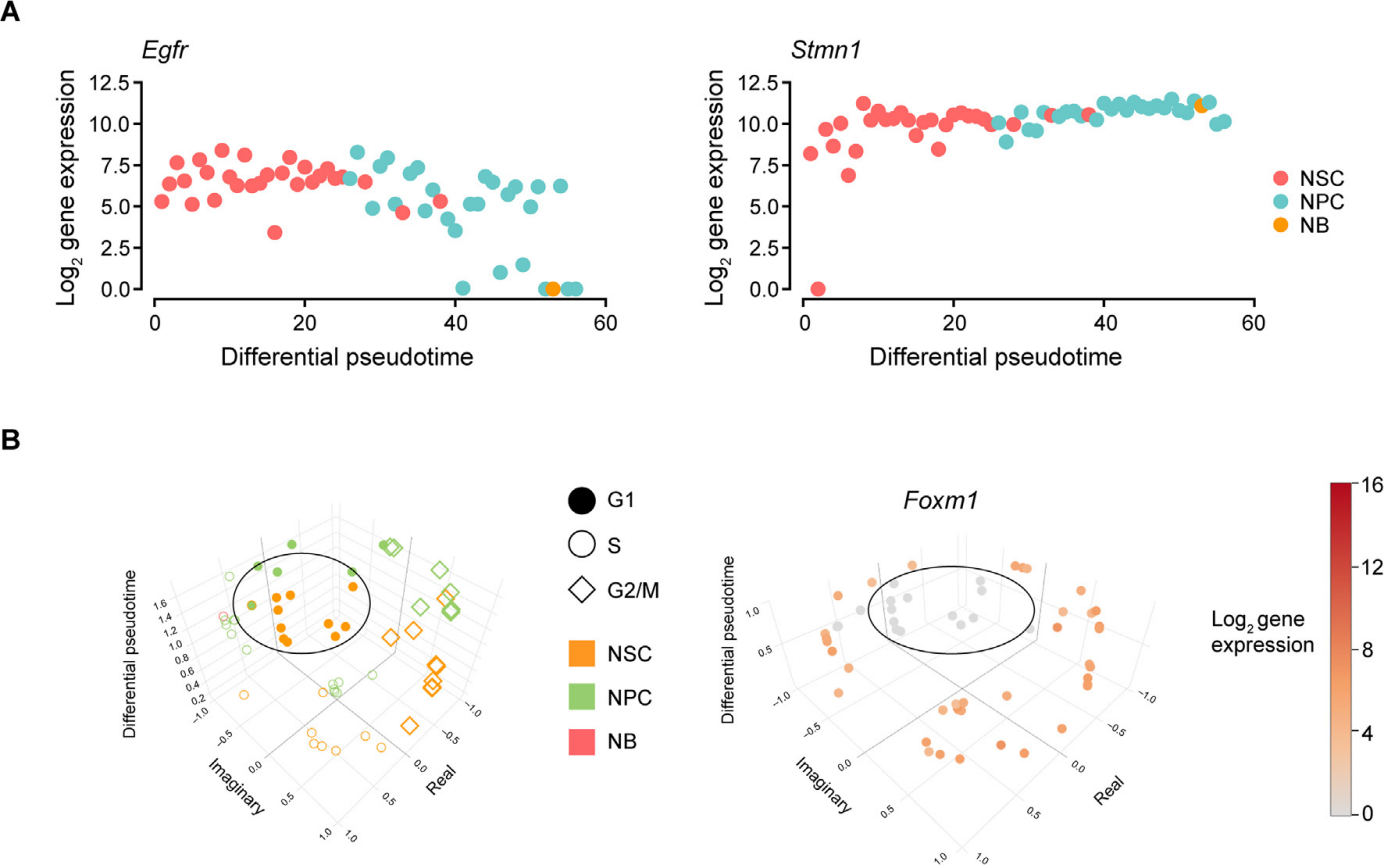
**Incorporation of cell proliferation and differentiation** **A.** We pre-processed the NSC-Llorens dataset by removing G0-like cells and calculated the pseudotime on the remaining cells. Expression changes of *Egfr* and *Stmn1* were plotted against the inferred differential pseudotime. **B.** Left panel, depiction of both cell types and cell cycle stages on the same plot. The vertical axis represents the differential pseudotime; the imaginary and real axes represent the proliferation pseudotime. Right panel, the expression value of *Foxm1* for each cell.

As an attempt to visualize the relationship between the cell cycle process and differentiation, 3D plots are produced for the NSC-Llorens dataset. We removed G0-like cells from the dataset before exploring the relationship between cell proliferation and differentiation. The developed approach was run once to remove all G0-like cells from the dataset (89 cells with *P* < 0.001 were removed). The differential pseudotime was re-calculated with redPATH on the remaining cells, and cell cycle analysis results were obtained by running reCAT. Here, redPATH (CI: 0.868, BI: 0.973, KC: 0.832) outperforms SCORPIUS (CI: 0.849, BI: 0.768, KC: 0.566) on the remaining 56 cells, showing its reliability even in a very small dataset. NSC marker genes (*Egfr* and *Stmn1* [7], [42]) further validated that most G0-like cells have been removed from the downstream analysis, where neither is expressed much during the quiescent state (Figure 6A).

Using the two statistics, dCor and MIC, we uncovered three genes (*Foxm1*, *Tubb5*, and *Nek2*) with a threshold of 0.65. These genes highly correlate with both cell proliferation and differentiation. Differentially associated marker genes such as *Dcx*, *Dlx1-2*, *Dlx5*, *Tubb3*, *Cd24a*, *Sox11*, *Dlx6as1*, *Mfge8*, *Sp9*, and *Atp1a2*, are in concordance with previous studies [6], [7], [47], [50]. Similarly, we also uncovered interesting genes that are cell cycle-related, such as *Cdk1* and *Aurkb*, which associate with both cell proliferation and NSC activation.

*Foxm1* was recently reported to regulate a microRNA network that controls the self-renewal capacity in NSCs [51]. redPATH provides an interactive plot that can visualize different cell types, cell cycle stages, and gene expression together. Focussing on NSCs and NPCs in Figure 6B (points outside the ellipse), we noted that *Foxm1* is highly expressed in S and G2/M cycling stages, indicating cell proliferation. This further confirms the conclusion that the high expression of *Foxm1* promotes the self-renewal capacity of NSCs. Observing the NSCs within the ellipse (the inner orange points on the left), these cells are lowly expressed compared to the outer NSCs. The results suggest that these NSCs may be at their earlier activation stages, which are more quiescent-like than the highly expressed activated NSCs.

### redPATH analysis on glioma datasets

In the analysis of cancer datasets, we assumed that the snapshot of data provides the different development stages of single cells among the dissected tissue. We are particularly interested in the progression change in gene expression from microglia/macrophages to malignant cells within a tumor dissection. Normal microglia cells exist to eliminate any intruding cells, also acting as antigen-presenting cells that activate T cells [52]. However, immune functions of microglia/macrophages within glioma tumors are often impaired. These cells are more commonly known as GAMs, which regulate tumor growth [9], [10], [12], [13]. As the original publication [5] suggests, malignant cells include some properties of NSCs with active differentiation in glial cells specifically. Although the tumor microenvironment is much more complicated, we can infer gene modules and possible relevant genes.

#### *Gene module extraction*

In the original publication [5], the authors used clustering and copy number variation analysis to classify each cell in the tumor microenvironment (malignant cell, microglia/macrophage, oligodendrocyte, and T cell). Although these four cell types do not differentiate into one another, GAMs and T cells are altered to regulate malignant cells. Here, we re-ordered the cells using redPATH and successfully recovered a pseudo developmental trend to observe the gene expression change.

**Figure 7:**
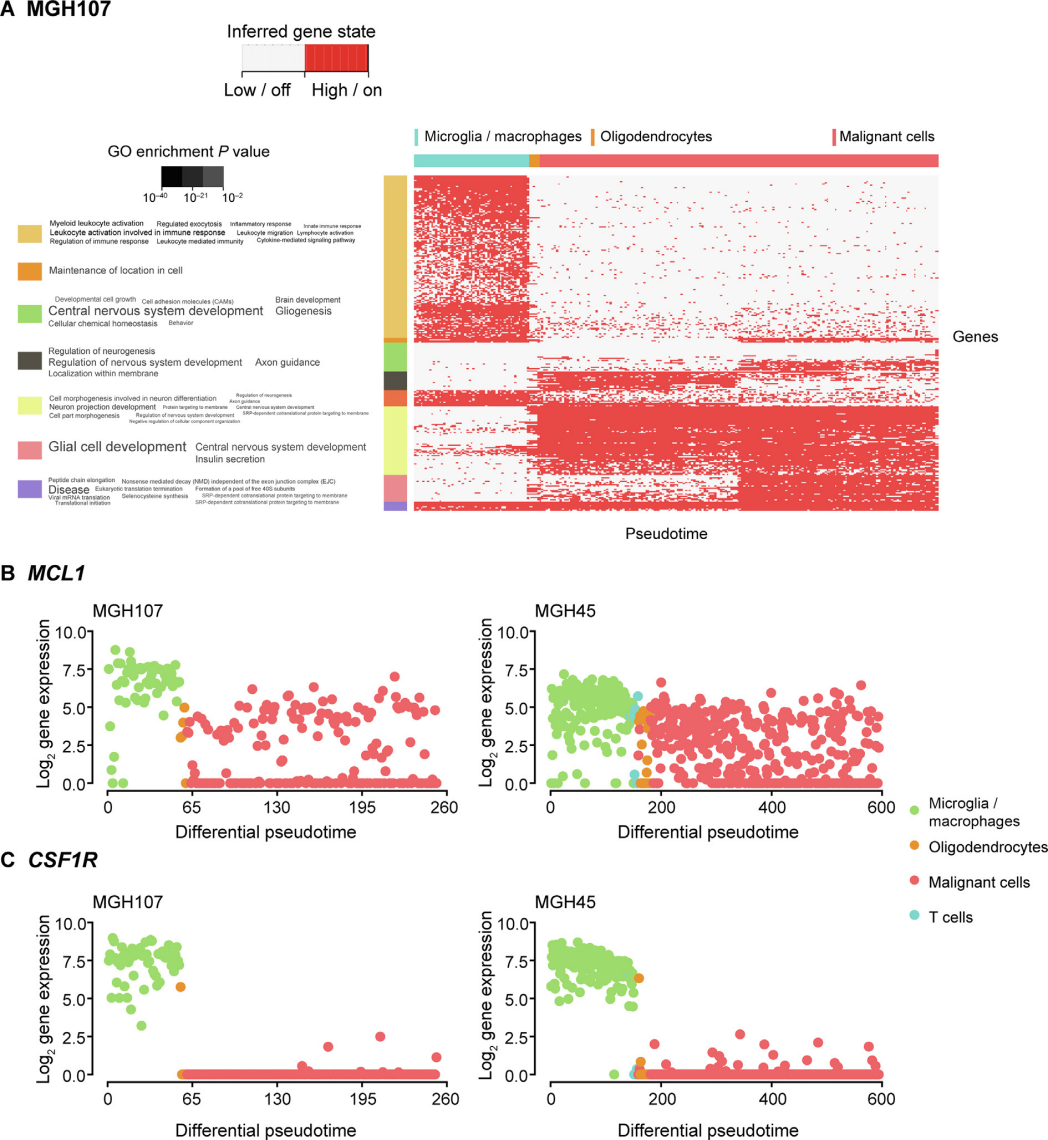
**Analysis of glioma datasets** **A.** Heatmap plot for patient MGH107 was produced according to the inferred HMM results from redPATH, indicating on/highly expressed state or off/lowly expressed state of each gene. The horizontal ordering denotes the differential pseudotime, while each row represents a significantly identified gene. The gene clustering result is shown on the left with GO enrichments (*P* < 0.01). **B.** Expression trend of *MCL1* along the pseudotime for MGH107 and MGH45. **C.** Expression trend of *CSF1R* along the pseudotime for MGH107 and MGH45. HMM, hidden Markov model.

**Supplementary Figure S7:**
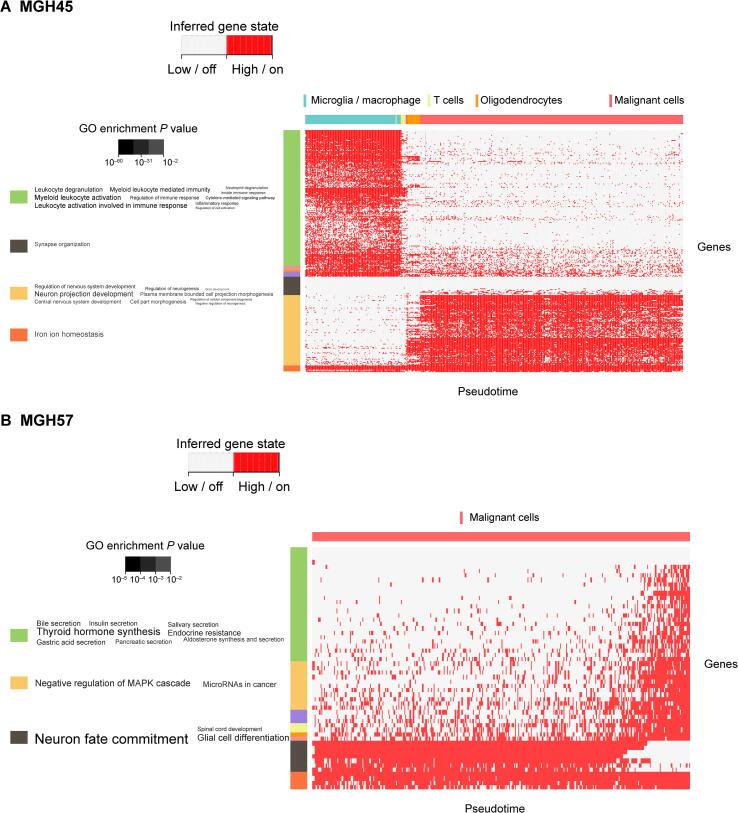
**Heatmap analysis on glioma datasets**. Heatmap plots for MGH45 (**A**) and MGH57 (**B**) were produced according to the inferred HMM results from redPATH, indicating an on/highly or off/lowly expressed state of each gene. The horizontal ordering denotes the differential pseudotime, while each row represents a significantly identified gene. The gene clustering result is shown on the left with Gene Ontology enrichments (P < 0.01).

MGH107 is a grade II astrocytoma that has not been treated yet. In Figure 7A, we observed a gradual change in gene expression and identified two distinct subpopulations of malignant cells in MGH107. The other two grade IV tumors showed less progression but still revealed a subpopulation in MGH57 (Figure S7). Using dCor and MIC, we retained 921, 55, and 762 significantly identified genes for analysis for MGH45, MGH57, and MGH107, respectively (threshold ≥ 0.5). We observed that the gene expression profile of oligodendrocytes is closer to a subpopulation of malignant cells (Figure 7A).

#### *Stem-cell like subpopulation*

**Supplementary Figure S8:**
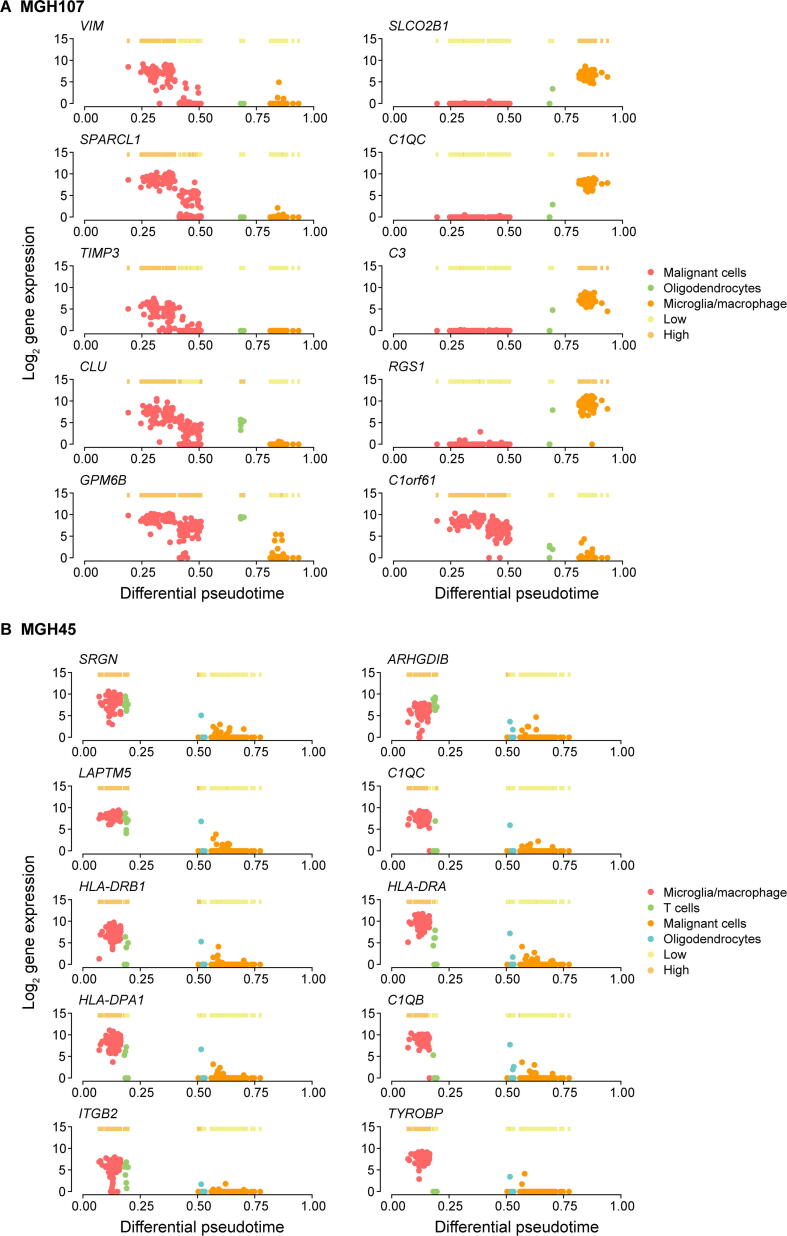
**Top-ranked significant genes from glioma datasets**. Ten top-ranked genes were selected using dCor and MIC for MGH107 (**A**) and MGH45 (**B**). Each gene expression is plotted against the calculated pseudotime with the inferred gene state. The cell type information is also reflected in the coloring of the points. dCor, distance correlation; MIC, maximal information coefficient.

Focussing on the ‘‘glial cell development / central nervous system development” gene module of MGH107 in Figure 7A, astrocytic and stem cell-like markers (*ATP1A2*, *GFAP*, *CLU*, and *ALDOC* [5], [42], [53]) were found to be expressed in the latter half of the malignant cells. In contrast, quiescent markers such as *ID3* remained silenced. Additionally, a subpopulation of malignant cells can be identified by observing the top-ranked significant genes such as *VIM*, *SPARCL1*, and *TIMP3* (Figure S8). The results indicate a high potency of the malignant cells to differentiation and proliferation. The malignant cells of MGH45 (grade IV recurrent glioblastoma) showed a constant gene expression pattern. However, MGH57 (grade IV glioblastoma) revealed a relatively small subpopulation of malignant cells that do not express *OLIG1*, *OLIG2*, *DLL1*, *CCND1*, and *IGFBPL1*, but express *ALDOC* and *ATP1A2* (File S1; Figure S7). Here, *ATP1A2*, *IGFBPL1*, and *ALDOC* are possible significant stem cell-like markers identified from the aforementioned analysis of the NSCs. These results indicate a subset of non-proliferative malignant cells in MGH57 and MGH107. MGH45 is from a recurrent glioblastoma patient; hence, a large portion of malignant cells may be stem cell-like.

#### *Apoptosis program in gliomas*

An interesting exploratory finding is the apoptosis program within gliomas. Apoptosis is a mechanism within the body that is activated intrinsically or extrinsically, which leads to cell death. All three tumor patients had not been treated with medication or radiation before; hence external factors of cell death are not applicable.

*MCL1*[54], [55], [56], a critical BCL-2 family apoptosis regulator, is significantly expressed within the gene cluster of “glial cell development” (dCor: 0.59; MIC: 0.50) of MGH107. The expression of *MCL1* activates *BAX* and *BAK* modules in the apoptosis pathway in general. Recent research [56] has shown that silencing *MCL1* leads to inhibition of cell proliferation, thereby promoting apoptosis in glioma cells. Here, we observed two subpopulations of malignant cells in MGH107 (Figure 7B). Interestingly, *MCL1* is highly expressed in microglia/macrophages of MGH107 and MGH45, promoting cell proliferation of the immune system. Comparing the number of malignant cells which possibly promote apoptosis to the total number of malignant cells, we observed similar proportions across different tumors: MGH107 (0.45), MGH57 (0.5), and MGH45 (0.35).

Intuitively, MGH45 appears to be a severe case with a smaller number of malignant cells that activate apoptosis. This confirms with the tumor grading of MGH45 (IV). Although numerous other apoptosis signaling pathways are available, further biological validation would be beyond the scope of this analysis. Drugs targeting the BCL-2 family and MCL1 inhibitor were under pre-clinical trials in 2015 with promising results [11], [57], [58].

#### *Discovery of potential target genes*

Additionally, we plotted some of the top marker genes in Figure S8. There are numerous overlaps in MGH45 and MGH107, where *CSF1R* (dCor: 0.95 and MIC: 0.78 for MGH45; dCor: 0.93 and MIC: 0.78 for MGH107) is discovered with distinct change between microglia/macrophages and malignant cells. It has been previously reported that inhibition of *CSF1R* in macrophages may lead to a reprogramming of macrophages, reducing tumor growth [14], [59]. However, experiments also showed that inhibition of *CSF1R* eventually acquires resistance, and PI3K signaling pathways are activated to support malignant cells [15]. It is trivial that the microglia/macrophages are overly expressed within the tumor microenvironment (Figure 7C).

Overall, redPATH can analyze single-cell transcriptome datasets with and without cell type labeling. As shown in the heatmap analysis of glioma cells, redPATH can also correctly recover the cell type segmentation along a developmental pseudotime.

## Discussion

With the initial intent to analyze pseudo developmental processes of single-cell transcriptome data, we developed a novel comprehensive tool named redPATH. We provided computational analytics for understanding cell development as well as cancer mechanisms. We showed the robustness of redPATH in recovering the pseudo developmental time of cells and its capability in detecting both branched and linear progressions. The algorithm demonstrates high consistency across different sample numbers as well as different feature selection methods. Subsequently, we implemented downstream analytical functions including 1) detection of G0-like cells, 2) gene discovery using dCor and MIC, 3) 2- or 3-state HMM segmentation inferring lowly or highly expressed gene state, 4) identification of branched or linear cell development, and 5) gene module extraction and 3D visualization for differentiation and proliferation processes.

In this study, we showed that redPATH could successfully recover the cell developmental processes, and we analyzed glioma datasets with a new perspective. We discovered stem cell-like and apoptotic marker genes (such as *ATP1A2*, *MCL1*, *IGFBPL1*, and *ALDOC*) along with a deepened understanding of diseases and cell development. We discovered significant novel genes using the pseudotime rather than testing the differential genes by groups. Although the advantage is that cell type labeling is not required here, this approach may fail when the pseudotime results perform poorly.

redPATH further attempts to visualize the coupling relationship between cell proliferation and differentiation. However, integrative models are preferred to analyze such processes simultaneously. The underlying mechanism remains obscure and requires more integrative computational models.

## Code availability

redPATH is available at https://github.com/tinglabs/redPATH.

## CRediT author statement

**Kaikun Xie:** Conceptualization, Methodology, Software, Formal analysis, Data curation, Visualization, Writing - original draft, Writing - review & editing. **Zehua Liu:** Conceptualization, Methodology, Formal analysis. **Ning Chen:** Conceptualization, Methodology, Supervision. **Ting Chen:** Conceptualization, Methodology, Writing - review & editing, Supervision, Funding acquisition. All authors read and approved the final manuscript.

## Competing interests

The authors have declared no competing interests.

## Supplementary material

Supplementary File S1 **Additional evaluations on redPATH and results for biological analysis**.
