# Supplementary material for: redPATH: Reconstructing the Pseudo Development Time of Cell Lineages in Single-cell RNA-seq Data and Applications in Cancer
Source: Genomics Proteomics Bioinformatics. 2021 Feb 17;19(2):292–305. doi: 10.1016/j.gpb.2020.06.014 (PMC8602773; doi:10.1016/j.gpb.2020.06.014)
Supplement: Supplementary File S1 — Additional evaluations on redPATH and results for biological analysis. [file mmc1.docx]

**File S1 Additional evaluations on redPATH and results for biological analysis**

**Modeling cell differentiation by asymmetric distance (KL-distance)**

We proposed to model the cell differentiation process by using KL-distance combined with a consensus Hamiltonian path algorithm. The intuition is that the asymmetrical property of KL better reflects the direction of differentiation development. We believe that it can capture biological information better. A comparison between the Euclidean distance and KL distance is made on subset data from four single-cell datasets (Figure S1). We sampled 8 cells, 20% of the cells, and 80% of the cells 50 times and conducted the evaluation. Brute force search is performed on a subset of 8 cells, and redPATH is performed on the other two subsets.

On the left panel of Figure S1B (NSC-Llorens), it is quite clear that the shortest path (calculated by brute force search) from KL-distance achieves a significantly better evaluation. In Figure S1A, S1 C−D, KL-distance achieves at least as good as Euclidean distance in worst-case. As N increases, the results become more stable across all four datasets.

**Identification of G0-like cells**

To date, there is no existing method to identify G0-like cells. Here we provide a simple statistical test to identify possible G0 cells in different datasets. The results are validated by using the NSC-Dulken dataset, where quiescent state NSCs (qNSC) have been labeled. qNSC cells are inactive stem cells that retain the ability to proliferate and differentiate upon activation.

First, we calculate the mean scores of each dimension (namely G1, S, G1/S, G2, M, and G2/M) of cell cycling genes. We then perform *k*-means clustering (*k* = 5) on the 6-dimensional mean scores to identify cell clusters. The distribution of each cluster is shown in Figure S2A-B for NSC-Llorens and NSC-Dulken, respectively.

By applying a simple ANOVA test to each pairwise comparison, we identified that cluster 4 (Figure S2B) as possible G0 cells with a threshold of *P* < 0.001 across all comparisons. Figure S2C further confirms our approach as most of the qNSC cells are included in this cluster, with little cells from aNSC and NPC. A small portion of aNSC is identified as G0-like, possibly due to an early stage of activation. Neural progenitor cells (NPC) contained both proliferative and in-active cells; here, only 3 out of 29 were identified as G0.

This procedure is repeated until one or more ANOVA tests become insignificant. The NSC-Llorens data analyzed in the main article was performed once to eliminate most of the G0-like cells.

**Comparison of feature (gene) selection**

Different gene selections will produce different results, especially for algorithms that include a dimensionality reduction step (such as SCORPIUS, TSCAN, Monocle2). Hence, we compared the performance of Monocle2 and TSCAN on their respective feature selection procedures and GO selected genes from redPATH. Results are shown in Figure S3A on four single-cell datasets. The performance is significantly better in NSC-Llorens and NSC-Shin when using GO selected genes in Monocle2 and TSCAN, while the results are relatively similar on NSC-Dulken and HSC-Schlitz (Figure S3B).

Additionally, we also compared the performance of redPATH using different gene selection methods (Figure 3C). We evaluated redPATH 20 times on both GO selected genes and the top 1000 genes selected from dpFeature on six datasets. Although in most datasets, there is a drop in performance using dpFeature, the difference is mostly insignificant (as most achieved evaluation > 0.8). Interestingly, in the mESC dataset, redPATH managed to achieve better results with dpFeature. In conclusion, we believe that our algorithm is relatively stable with different gene selection methods and will perform well in most datasets.

**Additional marker genes comparison between different algorithms**

*Sox9* and *Apoe* are NSC markers, while *Ccna2* is a known marker for proliferation/cell cycle. Hence *Ccna2* can be used to distinguish between qNSC and aNSC. Identical to results shown in the main article, SCORPIUS performs equally well in both NSC-Dulken and NSC-Llorens datasets compared to redPATH. However, the developmental trend in NSC-Shin differs. As shown in Figure S4A-B, qNSC is supposed to be highly expressed while SCORPIUS begins with relatively low expressed NSC at the beginning. Similarly, a contradicting trend is also depicted in *Ccna2* (Figure S4C), violating the assumption of a linear development process from qNSC -> aNSC -> NPC -> NB.

**Heatmap analysis on MGH45 and MGH57**

Both MGH45 and MGH57 are WHO grade IV tumors. Within the malignant cell population, there is a distinct small subpopulation in MGH57, while the expression change is relatively constant in MGH45 (Figure S7). The subpopulation inhibits some marker genes in glial cell differentiation/neuron fate commitment, such as *OLIG1*, *OLIG2*, and *IGFBPL1*.

**Modified arbitrary insertion algorithm**

Given a graph **G**(V, E), where V is the vertices (or single cells in this case) and edges (KL distance between cells). A modification on the original algorithm [55] is given below:

1. Start with any arbitrary vertex $v_{i}$
2. Find vertex $v_{k}$ s.t. ${d(v}_{i}{,v}_{k})$ is minimized forming a tour $v_{i}- v_{k}- v_{j}$

*Selection Step:*

1. Select any arbitrary vertex $v_{k}$ currently not in sub-path

*Insertion Step:*

1. Find edge (*i, j*) in sub-path which minimizes

$${d(v}_{i}{,v}_{k})+ {d(v_{k}, v}_{j})- {d(v}_{i}{,v}_{j})$$

2. Insert $v_{k}$ between $v_{i}$ and $v_{j}$

Repeat the selection step and insertion step iteratively until the Hamiltonian path solution is calculated.
